# Supplementary material for: Crop management shapes the diversity and activity of DNA and RNA viruses in the rhizosphere
Source: Microbiome. 2022 Oct 24;10:181. doi: 10.1186/s40168-022-01371-3 (PMC9590211; doi:10.1186/s40168-022-01371-3)
Supplement: Supplementary file 2 — Additional file 1: Supplementary Figure S1. Overview of field site. Supplementary Figure S2. Phylogenetic assessment of ssRNA phage vOTUs. Supplementary Figure S3. Accumulation curves of vOTUs. Supplementary Figure S4. Summed mean relative abundance of Leviviricetes families. Supplementary Figure S5. Detection of active vOTUs. Supplementary Figure S6. Summed mean relative activity of rhizosphere-priming vOTUs. Supplementary Figure S7. Linear relationships between the number of active vOTUs 3 detected. Supplementary Figure S8. Variation in dsDNA vOTU activity explained by crop rotation 4 and soil compartment by recovery library. Supplementary Figure S9. Summed mean relative compartment-enriched viral activity. Supplementary Figure S10: Summed mean relative viral metabolic activity. Supplementary Figure S11. Beta diversity in bacterial community composition. [file 40168_2022_1371_MOESM1_ESM.pdf]

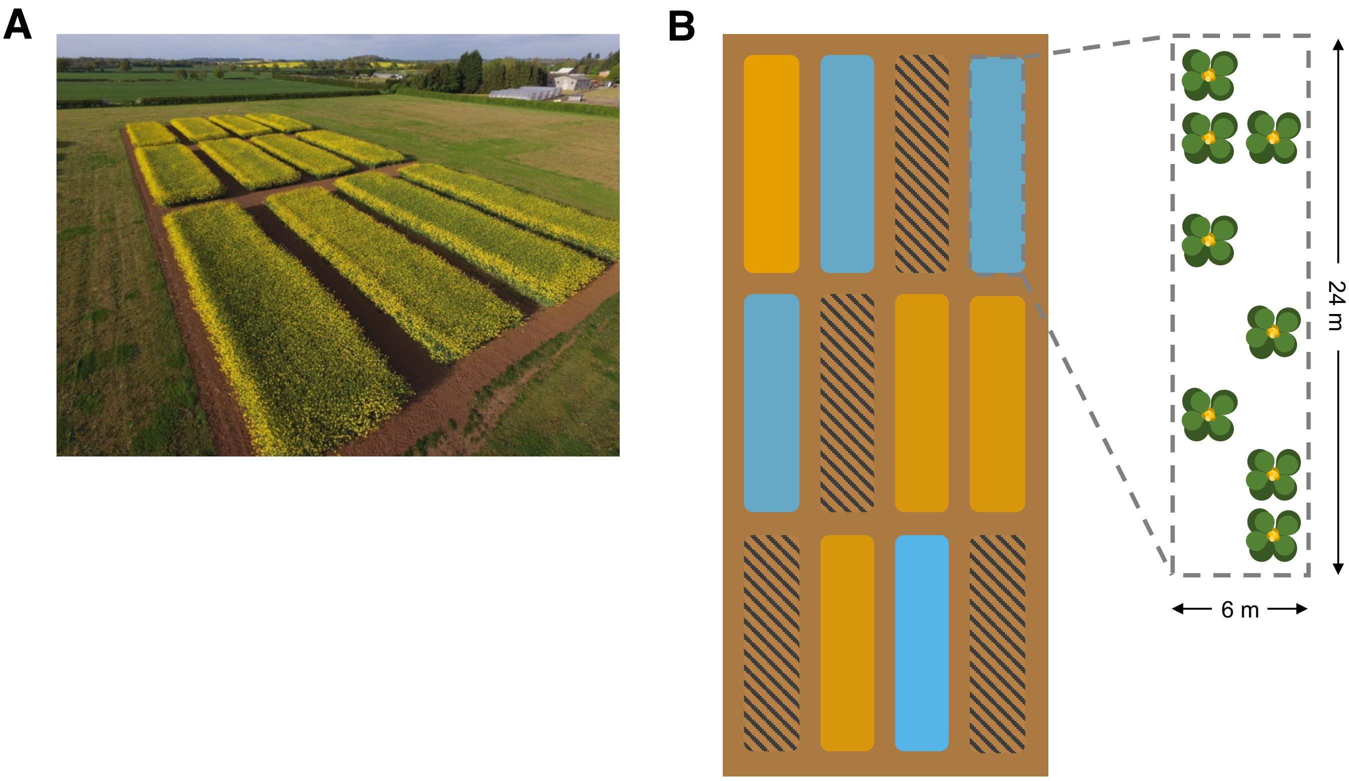

Supplementary Figure S1: Overview of field site. A Photograph of field site, including twelve plots, taken between stem extension and pre-harvest growth stages in the third year of the trial in 2017. **B** Schematic of field site, representing twelve plots. Colored plots indicate the two crop rotation practices sampled in this study: continuous cropping (orange) and virgin rotation (blue). Hashed plots were not sampled in this study. Expanded plot indicates that eight plants were sampled per plot to represent one replicate of each crop rotation practice.

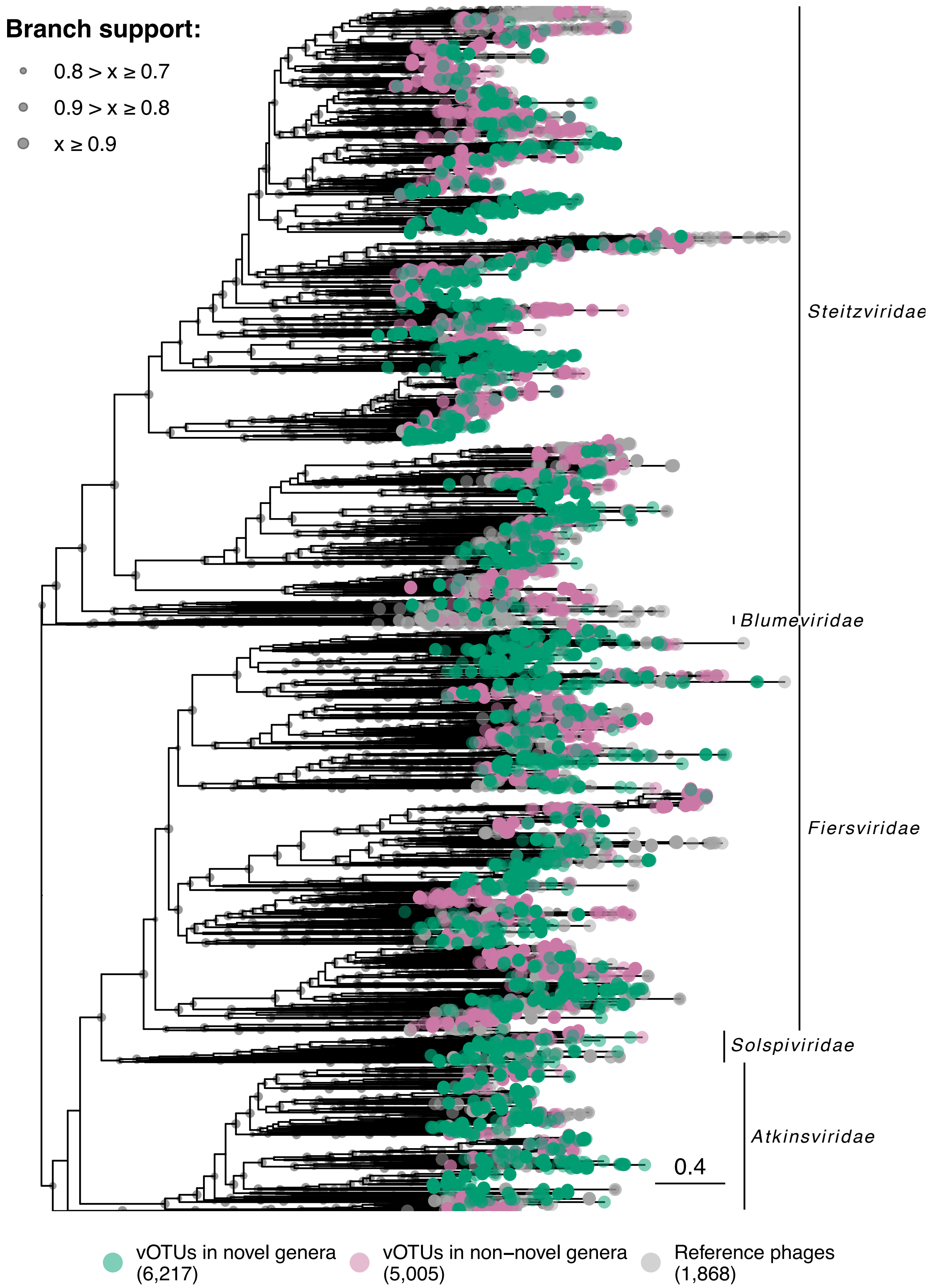
Branch support:
0.8 > x ≥ 0.7
0.9 > x ≥ 0.8
x ≥ 0.9
Steitzviridae
Blumeviridae
Fiersviridae
Solspiviridae
Atkinsviridae
0.4
vOTUs in novel genera (6,217)
vOTUs in non−novel genera (5,005)
Reference phages (1,868)

Supplementary Figure S2: Phylogenetic assessment of ssRNA phage vOTUs. Phylogeny of ssRNA phage vOTUs using concatenated core protein sequences (maturation protein, coat protein, and RNA-dependent RNA polymerase). Phylogenetic tree contains 1868 existing *Leviviricetes* phage sequences and our 11,222 full-length ssRNA phage vOTUs. Branch tip colours indicate novelty of genome sequence: vOTUs in novel genera (green, n labels indicate current *Leviviricetes* families. Brand node labels indicate branch support: ≥ 0.9 (large circles), ≥ 0.8 (medium circles), ≥ 0.7 (small circles), < 0.7 (no circle).

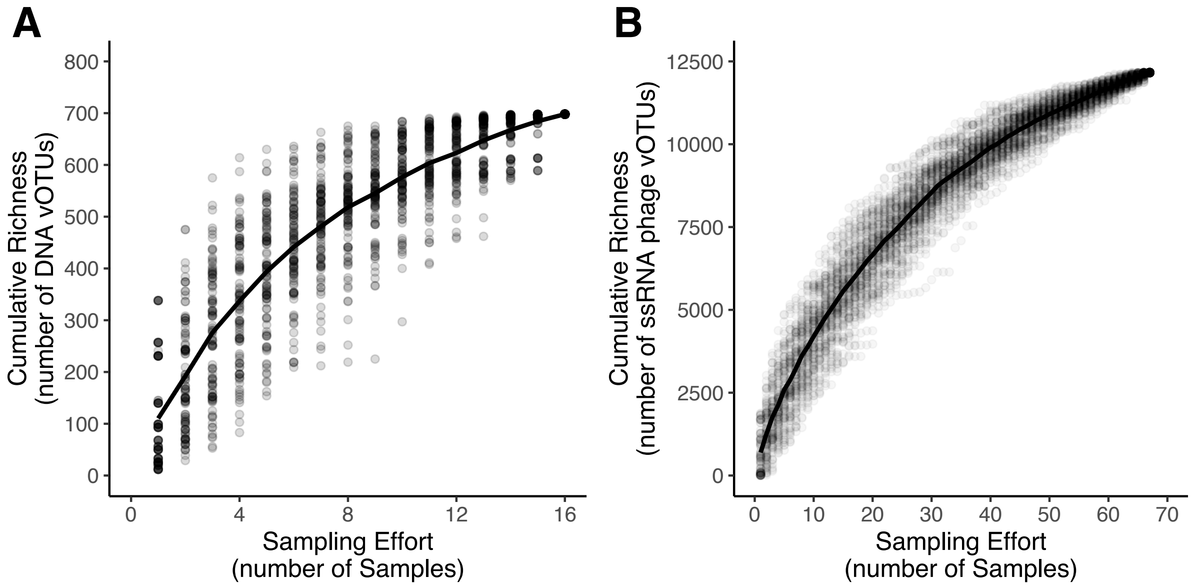

Supplementary Figure S3: Accumulation curves of vOTUs. Accumulation curves for **A** DNA vOTUs and **B** ssRNA phage vOTUs. Dots represent 100 permutations of cumulative richness at each sampling effort. Line indicates the mean cumulative richness in vOTUs detected.

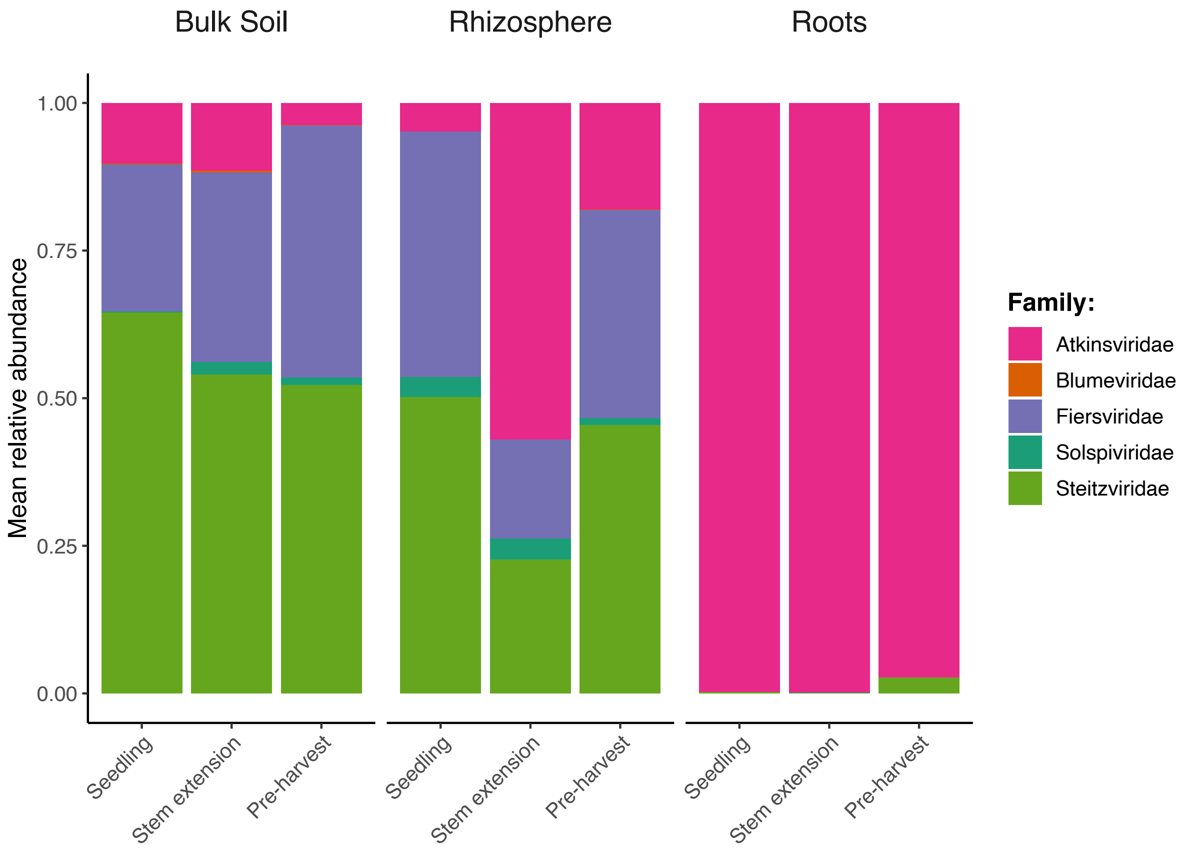

Supplementary Figure S4: Summed mean relative abundance of *Leviviricetes* families. Relative abundance of *Leviviricetes* families in each root/soil compartment, across growth stages. Colour indicates *Leviviricetes* family: *Atkinsviridae* (pink), *Blumeviridae* (orange), *Fiersviridae* (purple), *Solspiviridae* (blue-green), and *Steitzviridae* (green).

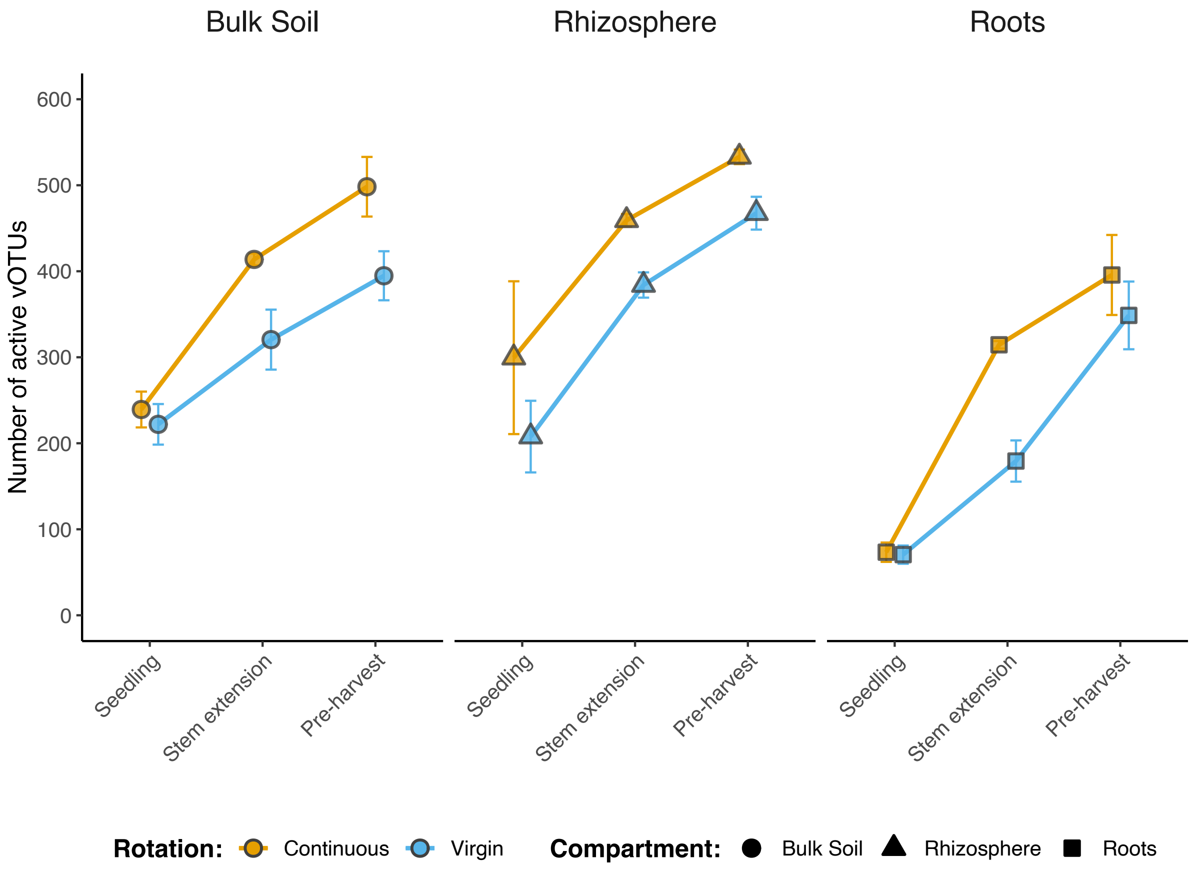

Supplementary Figure S5: Detection of active vOTUs. Mean number of active DNA vOTUs detected in each root/soil compartment, across growth stages. Shapes are colored based on field crop rotation strategy: continuous cropping (orange), virgin rotation (blue). Shapes indicate compartment: bulk soil (circles), rhizosphere soil (triangles), and roots (squares). Error bars denote a 95% confidence interval around the mean.

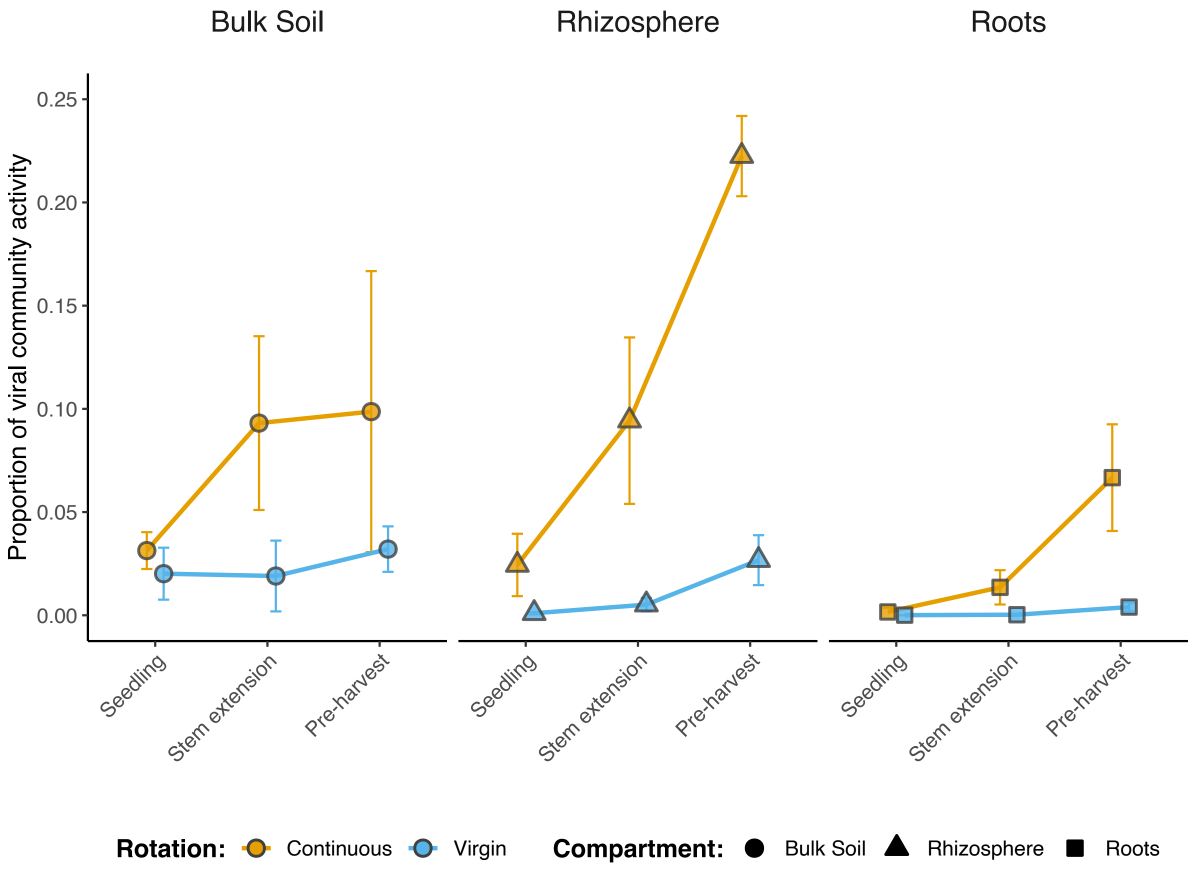

Supplementary Figure S6: Summed mean relative activity of rhizosphere-priming vOTUs. Mean proportion of viral community activity represented by rhizosphere-priming vOTUs (n = 196) in each root/soil compartment, across growth stages. Rhizosphere-priming vOTUs were detected in the seedling rhizosphere under continuous cropping but were absent in the seedling rhizosphere under virgin rotation. Shapes are colored based on field crop rotation strategy: continuous cropping (orange) and virgin rotation (blue). Shapes indicate compartment: bulk soil (circles), rhizosphere soil (triangles), and roots (squares). Error bars denote a 95% confidence interval around the mean.

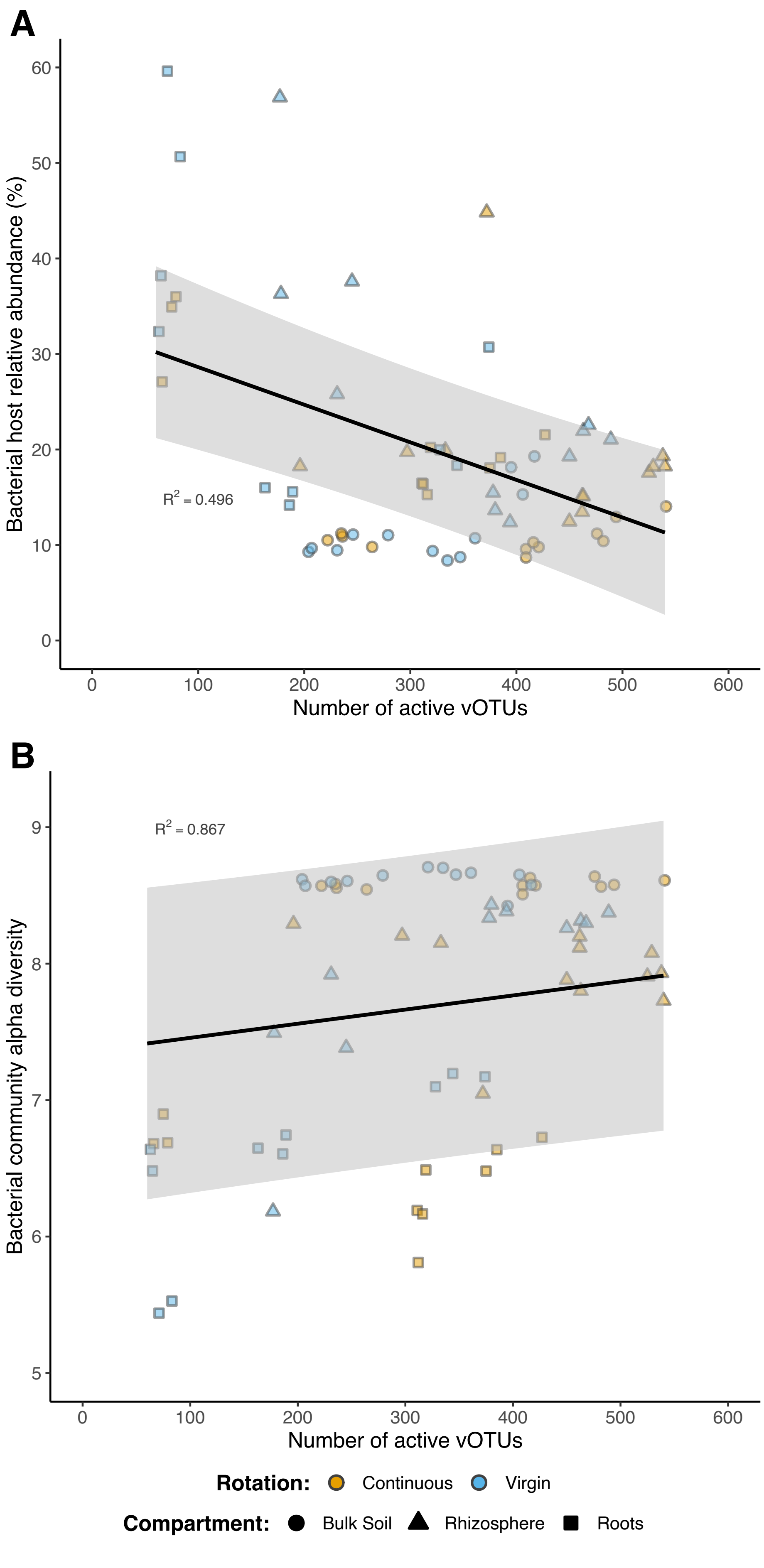

A
Bacterial host relative abundance (%)
Number of active vOTUs
$R^2 = 0.496$
0
10
20
30
40
50
60
0
100
200
300
400
500
600
B
Bacterial community alpha diversity
Number of active vOTUs
$R^2 = 0.867$
5
6
7
8
9
0
100
200
300
400
500
600
Rotation:
Continuous
Virgin
Compartment:
Bulk Soil
Rhizosphere
Roots

Supplementary Figure S7 - Linear relationships between the number of active vOTUs detected and A Summed host abundance, and **B** Bacterial community alpha diversity**.** Linear mixed effect models were run using compartment as a fixed effect. Line indicates model prediction, with gray cloud representing a 95% confidence interval around the predicted values. Shapes are colored based on field crop rotation strategy: continuous cropping (orange) and virgin rotation (blue). Shapes indicate compartment: bulk soil (circles), rhizosphere soil (triangles), and roots (squares).

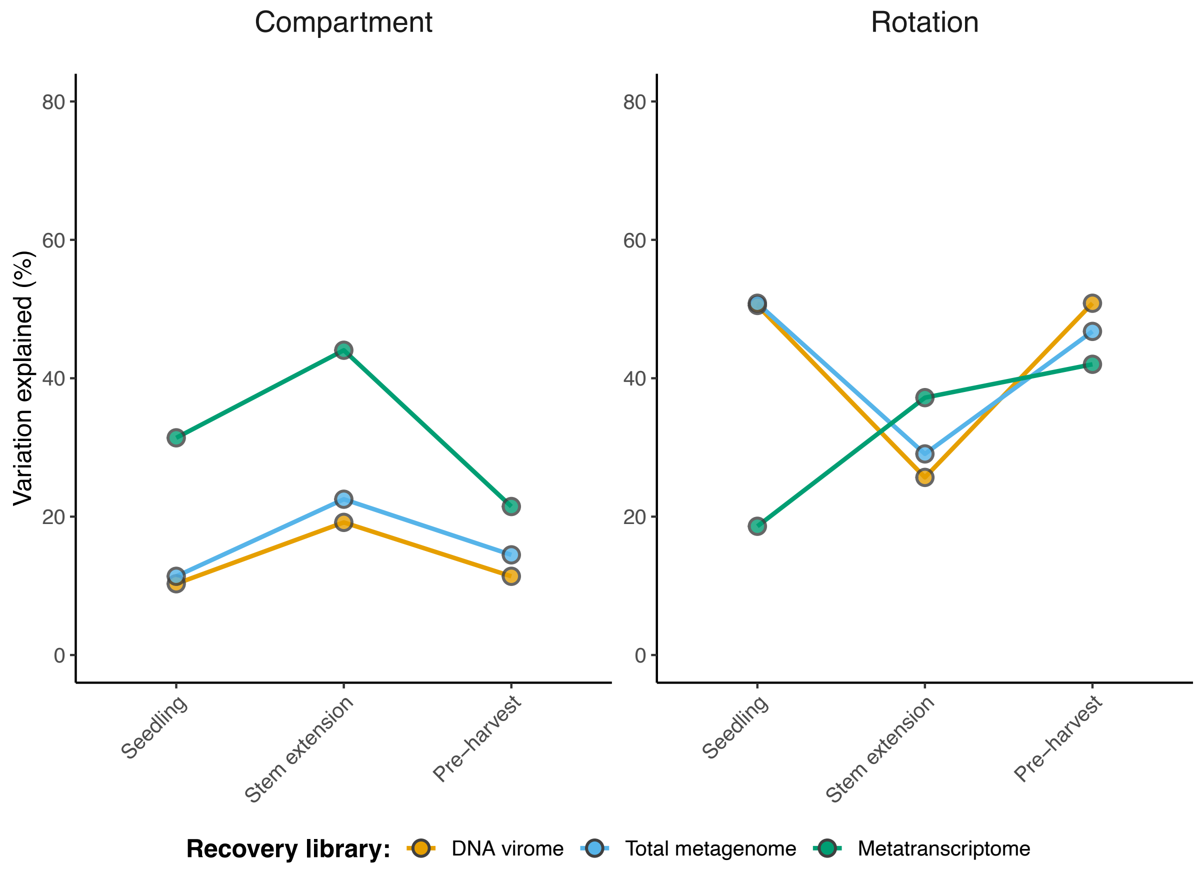

Supplementary Figure S8: Variation in dsDNA vOTU activity explained by crop rotation and soil compartment by recovery library. PERMANOVA results describe the variance explained by crop rotation and soil compartment, respectively, across growth stages. Points are colored based on viral activity from vOTUs recovered from the DNA virome (orange; seedling, n = 1741; stem extension, n = 1465; pre-harvest, n = 3140), total metagenome (blue; seedling, n = 368; stem extension, n = 1379; pre-harvest, n = 1772), and metatranscriptome (green; seedling, n = 3844; stem extension, n = 5078; pre-harvest, n = 5653).

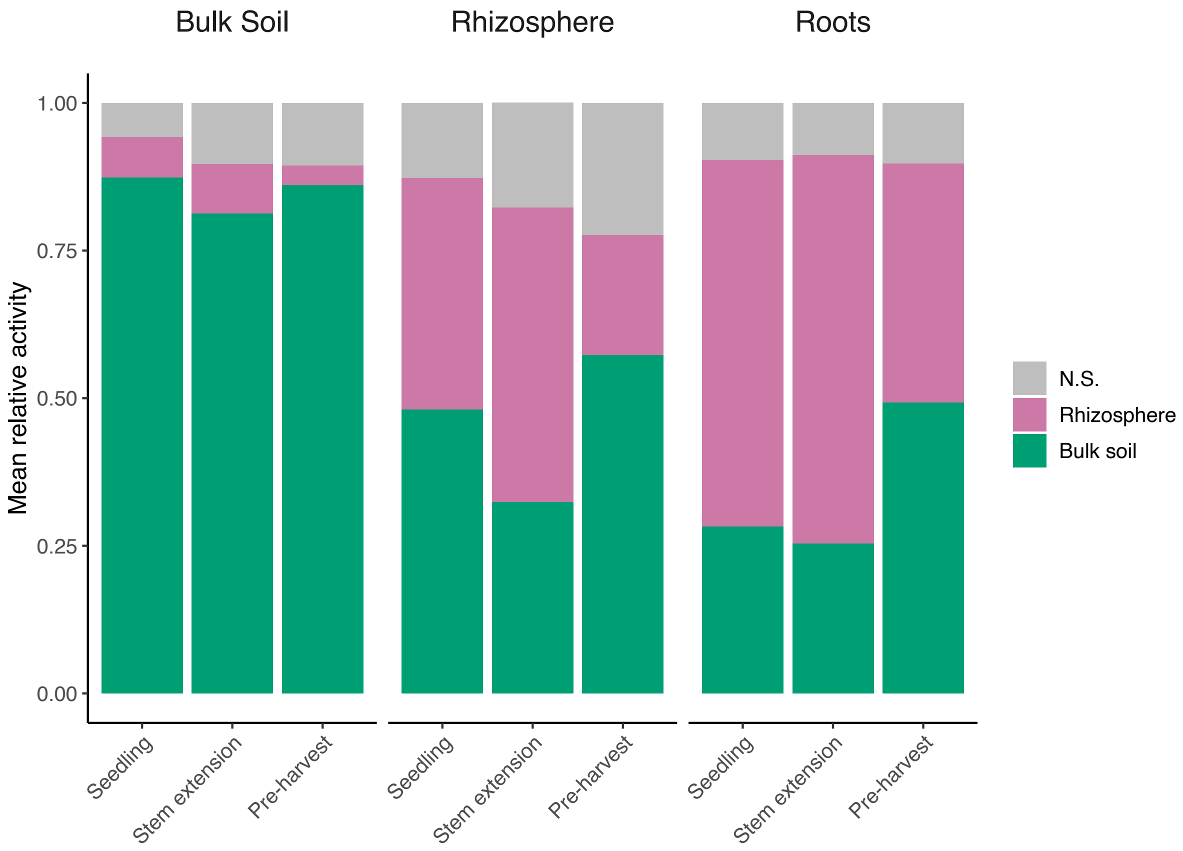

Supplementary Figure S9: Summed mean relative compartment-enriched viral activity. A Relative compartment-enriched viral activity in each root/soil compartment, across growth stages. Colour indicates soil compartment enrichment: N.S. (non-significant; gray), in rhizosphere soil (pink), and in bulk soil (green).

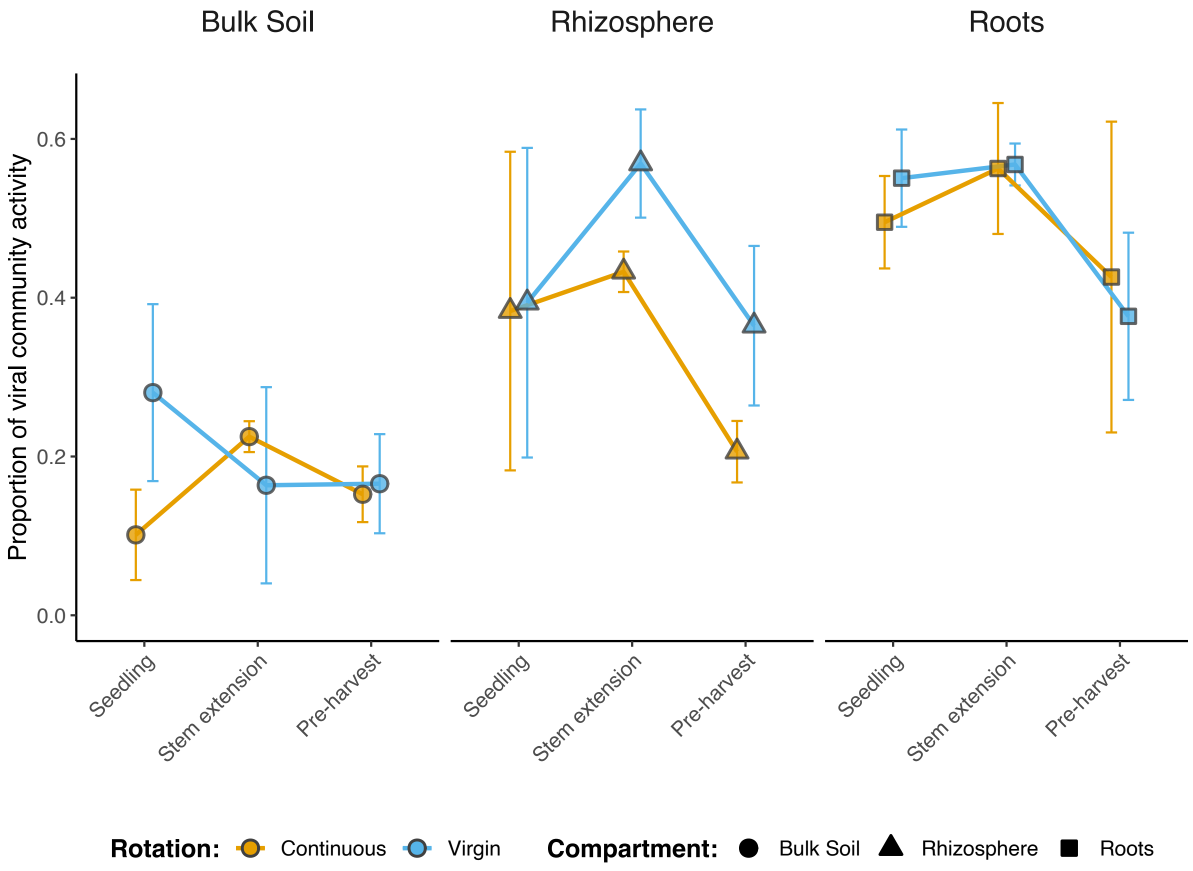

Supplementary Figure S10: Summed mean relative viral metabolic activity. Proportion of viral community activity represented by viral-encoded metabolic genes in each root/soil compartment, across growth stages. Shapes are colored based on field crop rotation strategy: continuous cropping (orange) and virgin rotation (blue). Shapes indicate compartment: bulk soil (circles), rhizosphere soil (triangles), and roots (squares). Error bars denote a 95% confidence interval around the mean.

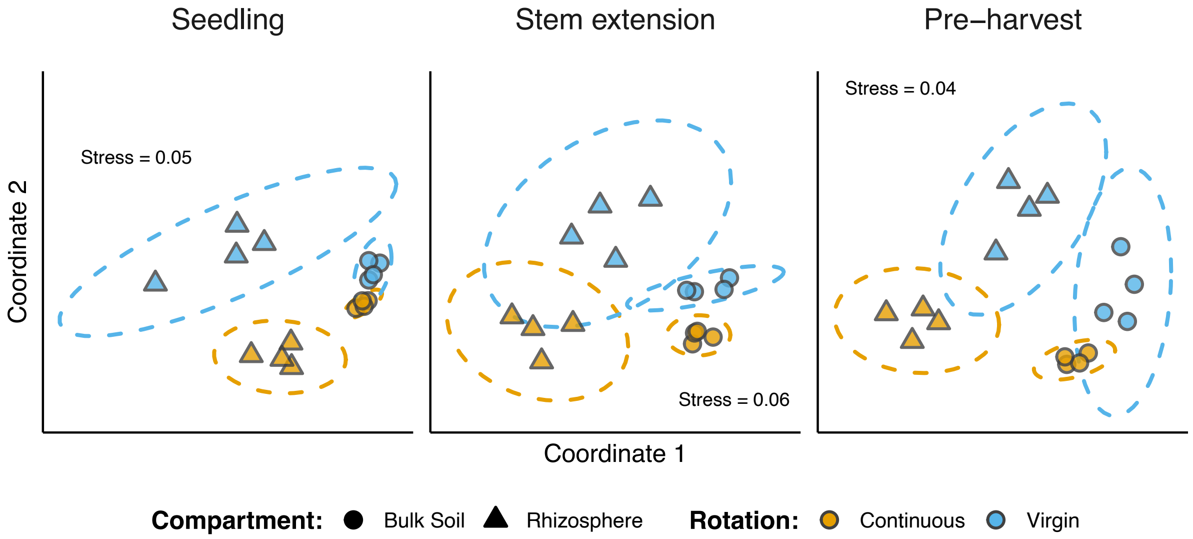

Supplementary Figure S11: Beta diversity in bacterial community composition. Non-metric multidimensional scaling (NMDS) ordination plots, representing the dissimilarities between community compositions, for each growth stage. Ordinations represent community compositions containing 16S rRNA gene OTUs at seedling (n = 27,335), stem extension (n = 28,235), and pre-harvest (n = 28,958). Shapes are colored based on field crop rotation strategy: continuous cropping (orange) and virgin rotation (blue). Shapes indicate compartment: bulk soil (triangles) and rhizosphere soil (circles). Stress values associated with two-dimensional ordination are reported for each plot.
